# Dentin Microhardness and Sealer Bond Strength to Root Dentin are Affected by Using Bioactive Glasses as Intracanal Medication

**DOI:** 10.3390/ma13030721

**Published:** 2020-02-05

**Authors:** Renata Grazziotin-Soares, Letícia Gomes Dourado, Bruna Lais Lins Gonçalves, Diego Machado Ardenghi, Meire Coelho Ferreira, José Bauer, Ceci Nunes Carvalho

**Affiliations:** 1 Department of Restorative Dental Sciences, College of Dentistry, University of Florida, Gainesville, FL 32610, USAdardenghi@dental.ufl.edu (D.M.A.); 2 School of Dentistry, CEUMA University, São Luís, 65065-470, Brazil; leticiapgomes26@gmail.com (L.G.D.); brunalais25@hotmail.com (B.L.L.G.); meirecofe@hotmail.com (M.C.F.); 3 Discipline of Dental Materials, School of Dentistry, University Federal of Maranhão (UFMA), São Luis 5085-582, Brazil

**Keywords:** dentin, microhardness, obturation, push-out bond strength, niobium, bioactive glasses, intracanal medication

## Abstract

This study investigated the human dentin microhardness (MH) and the MTA Fillapex^®^ (Fillapex) and AH Plus^®^(AH) bond strength (BS) to dentin after using calcium hydroxide (Ca(OH)_2_) and bioactive glasses (45S5 and an experimental niobium phosphate bioactive glass (NbG)) as intracanal medications. For the MH test dentin slices were filled with medications and were submitted to Knoop MH (KHN) test (at day-0 (baseline data/without medication) and at day-15 (after using medication)). For the BS test, after medications had remained for 15 days in the roots, dentin slices were obtained and filled with the sealers. Seven days later, sealer BS to dentin was measured by push-out test (MPa). Data were statistically analyzed. Failure mode was visually assessed. The use of NbG, 45S5 for 15 days, increased the dentin MH and reduced the BS between AH sealer and dentin, but did not interfere with the Fillapex BS.

## 1. Introduction

In vitro studies have shown that using the intracanal medication calcium hydroxide (Ca(OH)_2_) negatively affects dentin properties, for instance, the dentin microhardness [1,2], and the root fracture resistance of extracted teeth [3,4]. This weakening effect in dentin is associated with the reported 32%–40% clinical incidence of root fractures in patients who were submitted to long-term treatment with calcium hydroxide (Ca(OH)_2_) intracanal medication [4,5].

Similarly, the use of calcium hydroxide Ca(OH)_2_ as intracanal medication can affect the bond strength of root canal sealer to dentin. The presence of remaining particles of Ca(OH)_2_ attached to the canal walls might affect the sealer setting [6]. This would increase the likelihood of filling material dislodgment, microbial (re)contamination and percolation of fluids from the coronal and apical paths [7].

Bioactive glasses are alternatives to Ca(OH)_2_ as intracanal medication. These biomaterials promote bone regeneration and dentin remineralization because they have the ability to form a layer of hydroxycarbonate apatite [8,9,10]. Bioactive glass has antimicrobial effects in closed systems [11] and differently than calcium hydroxide, the antibacterial efficacy of bioactive glass increases when it is mixed with dentin [11,12]. Furthermore, bioactive glasses release calcium, phosphate, sodium, silica and depending on liquid exchange in the environment, slowly transform into calcium phosphate [13]. More recently one study demonstrated that an experimental niobium phosphate bioactive glass (NbG) reduced the biofilm formed with multispecies having the lowest live percentage values after 14 days of use [11]. The mechanical properties of human root dentin are affected to a lesser degree when using bioactive glass suspension in comparison to calcium hydroxide [14].

The first bioactive glass described to form a bond with bone had the original composition 45S5 (45 wt% SiO_2_, 24.5 wt% CaO, 24.5 wt% Na_2_O, and 6 wt% P_2_O_5_) [15]. This phosphate glass bonded not to the bone and to soft connective tissues [16,17]. One of the disadvantages of the phosphate glasses is their reduced chemical durability in aqueous environments [18]. However, this can be improved with the addition of niobium oxide [19]. Researchers have shown that the addition of oxides into the bioactive glass improve its durability in aqueous environments [18,19], enhance its bonding ability to the tissues [17], as well as, improve its radiopacity and microhardness [20,21]. 

Considering the possibility to have an intracanal medication that acts in favor of mechanical properties of either dentin or root canal sealers, this present study focused on two different bioactive glasses used as intracanal medications: one commercially available (45S5) and the other an experimental composition (NbG). Therefore, we investigated the human dentin microhardness and the sealers (AH Plus^®^ and MTA Fillapex^®^) bond strength to dentin after using Ca(OH)_2_ and two bioactive glasses as medications. 

## 2. Materials and Methods 

The materials used as intracanal medications were: 1) 45S5 (SYLC^®^ Denfotex Research Ltd., Ilkley, UK), 2) experimental niobium phosphate bioactive glass (NbG), and 3) calcium hydroxide (Ca(OH)_2_, UltraCal**^®^**XS, South Jordan, UT, USA).

We obtained NbG by the fusion of chemical precursors in an electric furnace, according to previous studies [22,23]. The composition of the NbG was 44 wt% Nb_2_O_5_, 3 wt% P_2_O_5_, 21 wt% CaO, and 2 wt% Na_2_O, and 45S5 45 wt% SiO_2_, 24.5 wt% CaO, 24.5 wt% Na_2_O, and 6 wt% P_2_O_5_.

### 2.1. Preparation of the Roots

After approval by the local Research Ethics Committee (protocol number: 1.066.954/2015), we selected 112 palatal roots of first maxillary molars that were extracted for various reasons: 48 for the dentin microhardness (MH) analysis and 64 for push-out bond strength test (BS). 

We radiographed the roots to confirm the presence of a single root canal, the absence of calcification, resorption or previous endodontic treatment. Roots were cleaned and stored for 1 week in distilled water. Then, the roots were perpendicularly sectioned below the cementoenamel junction perpendicularly to their longitudinal axis using a cutting machine (Isomet 100 Precision Saw; Buehler Ltd., Lake Bluff, IL, USA), and the working length was visually determined at 1.0 mm short of the apical foramen. 

The canals were prepared using Reciproc instruments (VDW, Munich, Germany) up to a master file R50, with 10 mL of 1% sodium hypochlorite as irrigating solution. We removed the smear layer with 5 mL of 17% EDTA for 1 min. 

### 2.2. Treatment of the Samples and Dentin Microhardness (MH) Analysis 

To compare the mean MH values (KHN) between the groups, the following parameters were considered for the sample size: 95% level of confidence, power of 80%, standard deviation of five [4], and a minimum difference to be detected among groups of five points in the mean MH. The sample size required was 16. 

The roots were divided in groups in a random way, according to the materials that were used as medication (NbG, 45S5 and Ca(OH)_2_). Then, 3-mm thick cross-sectional slices (n = 16 per group) were obtained from the middle third of the roots by using a cutting machine (Isomet 100 Precision Saw (Buehler Ltd., Lake Bluff, IL, USA)) under constant water for cooling purposes. Slices were inserted into PVC tubes and acrylic resin. The dentinal surface of the slices was grinded in a polishing machine (Politriz, Arotec, Cotia, Brazil). 

Baseline MH was performed in a testing machine (Shimadzu HMV-2000, Kyoto, Japan) and a 40× Knoop diamond indenter, using 100 g loading for 15 s contact time. Each slice received three indentations on a distance of 20 µ from the canal lumen, in four different positions. Mean Knoop hardness values (KHN) were calculated for each specimen as the mean value of twelve indentations.

Subsequently, the medications were inserted into the lumen of the slices and over the sample, completely covering the dentin slice. Both bioactive glasses were mixed with distilled water in a 2:1 proportion. These values were predetermined in a pilot study using an analytic scale and double weighing method. The bioactive glasses (0.1 mL) were applied with a plastic syringe and NaviTips^®^ needles (Ultradent, South Jordan, UT, USA). Ca(OH)_2_ was used directly from the syringe provided by the manufacturer. At day 15, the medication was removed (the specimens were rinsed with distilled water and dried with soft absorbent paper) and MH was measured. 

### 2.3. Treatment of the Samples and Sealers’ Bond Strength to Dentin Analysis (BS)

We considered the following parameters for sample size calculation to compare the BS mean values (MPa) between the groups: 95% level of confidence, power of 80%, standard deviation of 2.5 [20], and a minimum difference to be detected among groups of four points in the BS value. The initial sample size required was six roots. Considering the losses probability, the final sample size adopted was eight.

The 64 roots were randomly divided in eight groups according to the medication (NbG, 45S5, Ca(OH)_2_, and control/no medication) and the sealer (AH Plus**^®^**(AH), Dentsply Maillefer, Ballaigues, Switzerland or MTA Fillapex^®^ (Fillapex) Angelus Indústria de Produtos Odontológicos S/A, Londrina, Brazil) as follows: 1) NbG + AH; 2) NbG + Fillapex; 3) 45S5 + AH; 4) 45S5 + Fillapex; 5) Ca(OH)_2_ + AH; 6) Ca(OH)_2_ +Fillapex; 7) control + AH; and 8) control + Fillapex.

The medications were inserted into the canals in the same manner (proportion, amount, and type of syringes used) as for the samples prepared for the MH test. At day-15, the medication was removed from the canal using the master apical file (R50), 5 mL of 1% sodium hypochlorite and 5 mL of EDTA. Each root was cross-sectioned into dentin slices. We cut-off and discarded the apical and cervical 2 mm of all specimens. The remaining root was cut into four 1.5-mm thick slices (± 0.01 mm). Each dentin slice was placed in single plastic containers immersed in physiologic solution at 37 °C for 48 h. 

The slices were dried, and the canals were filled with the sealer, by using a Centrix syringe (DFL, Rio de Janeiro, Brazil). The canal space was filled only with the sealer to achieve a better control of the failure. This eliminated the possibility to generate a systematic source of error as when two materials (sealer + core material) are used, which would create at least two interfaces to be “pushed” during the test. After filling the canal lumen with the sealer, the slices were covered with polyester strips. A glass slab rested over the samples to prevent sealer leakage out of the canal lumen. The specimens remained in an oven at 37 °C and in an environment with 100% of humidity for 30 days.

The sample unit was defined as each used roots. Thus, we calculated the arithmetic mean of the bond strength values of the test specimens that came from the same tooth.

**Figure 1:**
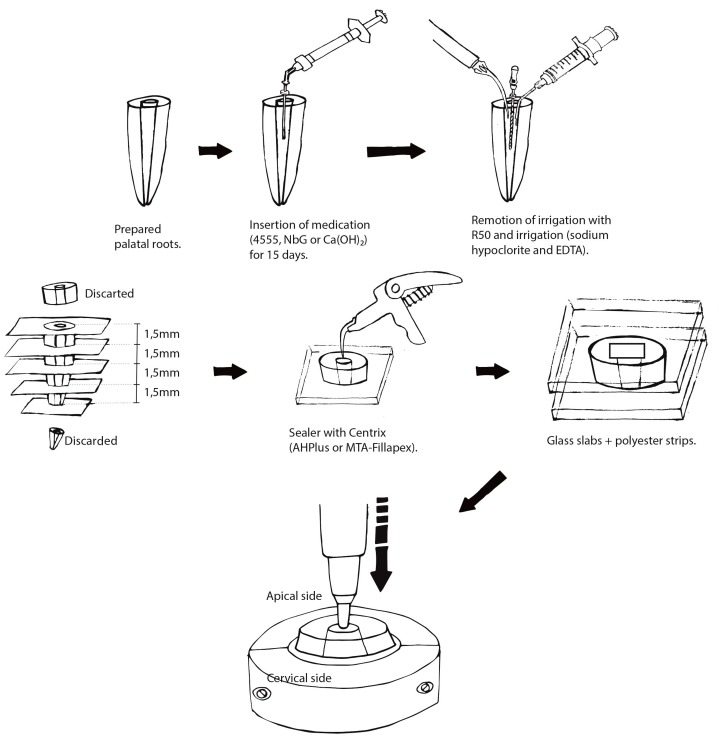
Schematic illustrating of the procedures of intracanal medication, root filling, and push-out test.

Cervical and apical images of the slices were captured using a digital camera (Q-Color 5; Olympus, America Inc., PA, US) attached to a stereomicroscopic loupe (SZ61; Olympus America Inc., PA, USA), under 40× magnification. Image J software (National Institute of Health, Bethesda, MD, USA http://rsb.info.nih.gov/ij/) was used to measure the lumen diameters of both sides of the slices. Sealer’s bond strength to dentin was measured under micropush-out test: The cervical surface of each tested specimen was placed on the support coupled to the base of the universal testing machine (EMIC, Instron Brasil Equipamentos Científicos Ltda, São José dos Pinhais, Brazil). The apical side was facing towards a cylindrical punch made of stainless steel that was fixed to a 50-kgf (kilogram-force) load cell. The selected post diameter was 0.2 mm smaller than the apical diameter of the slice; this prevented the post from touching the dentin walls during the test (Figure 1). The test was performed at a speed of 0.5 mm/min until complete extrusion of the filling, the value of the applied load was recorded by its abrupt drop in value. We calculated the bond strength values (MPa) using the load value in kgf—recorded after the tests—by dividing the maximum force necessary to displace the filling material in Newtons (N) by the area of the bond interface (BS = F/A), where: A = π(R + r)h^2^ + (R − r)^2^. A corresponded to the specimen’s surface area; π is the constant 3.1416; R is the largest radius of the cone; r is the smallest radius of the cone, and h is the slice thickness [24,25]. 

For the analysis of failure modes, the pushed-out specimens were cleaved longitudinally (buccolingual direction) and the root segments were observed under 10× magnification to measure the percentage of “free substrate” (dentin free of material): >75%: cohesive within the sealer, <25%: adhesive, >25% to <75%: mixed [25].

### 2.4. Data Analysis

For the MH data, statistical analysis was performed using the paired Student’s *t*-test to compare the experimental groups and their baseline measurements. The Shapiro–Wilk and Levene’s tests were performed to prove the data normality and the equality of the variances. To compare the percentage variation in MH among the groups, one-way ANOVA and Tukey’s were used. The level of significance was set at 0.05.

For the BS data, statistical analysis was performed using two-way ANOVA and post-hoc Tukey’s test. The size of the effect that the independent variables (type of intracanal medication and type of sealer) had on the BS was calculated with eta-squared (η^2^ partial). The level of significance was set at 0.05. 

All statistical tests were done using the software SigmaPlot 12.5 (Systat Software Inc., San Jose, CA, USA). The failure mode was assessed and reported using percentages (%).

## 3. Results

The results from MH analysis showed that Ca(OH)_2_ reduced the dentinal MH in 23.1% in relation to the baseline MH. Conversely, NbG and 45S5 induced an increase in the dentinal MH of 37.7% and 38.7%, respectively (Table 1).

Considering both root canal sealers, in the majority of the experimental groups, the sealer’s BS to dentin values were numerically lower when intracanal medication was used, in comparison to the control group. The BS values were higher for the AH sealer, in comparison to those samples obturated with Fillapex. The Fillapex BS values were not statistically different with or without the using of any previous intracanal medication. The AH BS values were higher for the control group (no previous intracanal medication) when compared to all other groups (*p* < 0.001) (Table 2).

The η^2^ partial values were 0.762 for the sealer and 0.367 for the intracanal medication, which means that 76.2% of variation in bond strength occurred due to the sealer, while only 37.7% occurred due to the intracanal medication. Failure mode results are shown in Table 3. In general, the majority of failure modes were cohesive.

## 4. Discussion

Our study showed that the experimental NbG and the 45S5 induced an increase in dentin MH to approximately 38% after 15 days of contact. In contrast, Ca(OH)_2_ reduced the dentin MH in 23% in relation to the baseline MH. The AH BS to dentin was lower when the root canal was previously filled with NbG (4.51 MPa), or with the other two medications, when compared to the control group (no medication) (11.33 MPa). The Fillapex BS to dentin was not influenced by the use of any intracanal medication.

Previous studies reported that the incorporation of Nb pentoxide increased the microhardness of the experimental adhesive materials [20,21]. Based on this, we questioned if the incorporation of Nb into the glass would improve the dentin MH. However, dentin MH and sealers BS to dentin were similarly affected by the two different bioglasses, either the experimental glass or the commercial glass.

The different mechanism of ions release between Ca(OH)_2_ and bioactive glasses can support the current results where we found a higher dentinal MH when bioactive glasses were in contact with dentin. In a previous study [23], it was shown that NbG and 45S5 release calcium ions, decreasing the amount over time (0 to 14 days), similarly to what occurs with Ca(OH)_2_. If we considered this fact alone, we should expect a reduction in dentinal MH for samples treated with bioactive glasses, in the same manner than that occurred for the samples treated with Ca(OH)_2_. However, in the previous study, the bioactive glasses release a much higher amount of sodium ions over time, in comparison to Ca(OH)_2,_ and they also start releasing phosphate ions at day-7, what did not occur for Ca(OH)_2_ [23]. These higher amounts of ions available in the first 14 days of using bioactive glasses could have improved mineral gain by the dentin in this study, consequently, improving hardness. 

Authors showed that dentin disks treated with bioactive glasses presented a smooth surface by crystalline formation, which could improve the sealing of dentinal tubules [9]. Despite morphology and analytical techniques being outside the scope of this present study, we can assume that a regular and smoother dentinal surface could be a factor contributing to our higher MH values. As EDTA was not used to wash the dentin slices before the day-15 MH measurement, it also may have contributed to a flatter surface, with covered dentinal tubules.

The present findings point that the use of bioactive glasses as intracanal medication could be a valuable option for clinical situations where the tooth may become weakened [3,4,5], such as, in apexification or endodontic regenerative procedures.

The bioactive glasses had similar influence to Ca(OH)_2_ on the sealers BS to dentin. Although, in this present study, only 37.7% of the variation in BS occurred due to the use of intracanal medications, it is worth highlighting that NbG, 45S5, and Ca(OH)_2_ induced a reduction in the bonding between AH and dentin. Ca(OH)_2_ reduces the dentinal permeability because it can block the dentinal tubules [26]. This blockage would impair the formation of sealer resinous tags on the entrance of dentinal tubules, consequently, reducing the bond strength on the filling material–canal wall interface. Another reason for a lower AH BS to root dentin could be the alkaline pH of NbG, 45S5, and Ca(OH)_2_ at day-15. A previous investigation showed pH of 7.3, 7.8, and 10.3 at day-14 for NbG, 45S5, and Ca(OH)_2_, respectively [23]. The alkaline pH somehow alters the morphology and/or the composition of the collagen matrix, impairing the filling material adhesion [27]. 

The blockage of tubules and the alkaline pH could be some of the reasons for the reduced AH BS; however, this reduction was not observed for Fillapex BS. Perhaps, the Fillapex BS was not affected by any of the intracanal medications because Fillapex is a bioceramic sealer (while AH is an epoxy resin-amine sealer). During the Fillapex setting reaction, the moisture present in the dentinal tubules hydrates the free calcium oxide, present in one of the pastes of the sealer, forming Ca(OH)_2_ which reacts with the salicylate, present in the other paste of the sealer, completing the setting [28]. The analogous compounds present in both, (in the medications and in the Fillapex-setting process) might have not affected the sealer BS to root dentin.

The failure mode assessment found high percentage of cohesive failures for the two tested root canal filling materials. These findings are in agreement with other studies [29] and can reinforce the idea that not only AH but also Fillapex presents some ability to adhere to canal walls, even if intracanal medication was previously used.

## 5. Conclusions

The findings from this study showed that the use of NbG, 45S5 for 15 days, increased the dentin MH and reduced the BS between AH sealer and dentin, but did not interfere with the Fillapex BS. These results point to a potential advantage to use bioactive glasses as intracanal medications, since they increased the MH of dentin and matched well with the MTA-based sealer. Clinically, these could be beneficial in situations where the tooth structure is weakened and in the improvement of sealer’s bonding.

## Figures and Tables

**Table 1:** Variation (percentage) in dentinal Knoop microhardness (KHN) values (mean ± standard deviation) after using intracanal medication for 15 days.

Intracanal Medication	Dentinal Microhardness		
	Baseline (day 0–no medication)	After Medication (day 15)	Variation (%)
NbG	34.7 ± 7.1 B	45.2 ± 11.2 A	+37.7 ± 37.6 a
45S5	35.4 ± 9.9 B	45.3 ± 8.7 A	+38.7 ± 48.8 a
Ca(OH)_2_	40.8 ± 4.9 A	29.5 ± 6.7 B	−23.1 ± 16.1 b

* Different capital letters in line indicate statistically significant differences between time-periods (paired Student’s *t*-test *p* < 0.05). Different lowercase letters in column indicate statistically significant differences between medications (ANOVA one-way *p* < 0.05).

**Table 2:** Sealers’ bond strength mean values (MPa) for AH Plus^®^ and MTA Fillapex^®^ according to the intracanal medication used for 15 days, previously to root canal obturation.

Intracanal Medication (15 days)					
		NbG	45S5	Ca(OH)_2_	Control (No Medication)
Sealer	AH Plus^®^	4.51^a^ (± 2.6)	7.14^a^ (± 3.9)	7.13^a^ (± 2.7)	11.33^b^ (± 3.5)
	MTA Fillapex^®^	0.33 (± 1.68)	0.15 (± 0.5)	0.97 (± 0.7)	0.38 (± 0.2)

* Different letters (a,b) in line indicate statistical difference (Post-hoc Tukey *p* < 0.05).

**Table 3:** Percentage (%) of failure modes (cohesive, adhesive, and mixed) considering the combination of intracanal medication (niobium phosphate bioactive glass (NbG), 45S5, Ca(OH)_2_, and control (no medication)) and root canal sealer (AH or Fillapex).

Groups	Adhesive (%)	Cohesive (%)	Mixed (%)
NbG + AH	10.15	71.9	18.65
45S5 + AH	5.7	70	24.3
Ca(OH)_2_ + AH	0	79.5	20.5
no medication + AH	0	71	29.0
NbG + Fillapex	0	83	17
45S5 + Fillapex	0	85	15
Ca(OH)_2_ + Fillapex	0	79	21
no medication + Fillapex	0	84	16
